# Supplementary material for: Gut Microbiota and Predicted Metabolic Pathways in a Sample of Mexican Women Affected by Obesity and Obesity Plus Metabolic Syndrome
Source: Int J Mol Sci. 2019 Jan 21;20(2):438. doi: 10.3390/ijms20020438 (PMC6358992; doi:10.3390/ijms20020438)
Supplement: Supplementary file 1 [file ijms-20-00438-s001.zip › Chavez-Carbajal et al IJMS-Supplementary Information/Chavez-Carbajal-MS&MB-Supplementary Information-File1-v10.pdf]

Gut Microbiota and Predicted Metabolic Pathways in a Sample of Mexican Women Affected by Obesity and Obesity plus Metabolic Syndrome

Alejandra Chávez-Carbajal[1], Khemlal Nirmalkar[1], Ana Pérez-Lizaur[2], Fernando Hernández-Quiroz[1], Silvia Ramírez-del-Alto[2], Jaime García-Mena[1]*, César Hernández-Guerrero[2]*

[1] Departamento de Genética y Biología Molecular, Cinvestav-IPN, Av IPN 2508, Ciudad de México, 07360, México. Phone: +52 (55) 5747-3800 Ext. 5328; alejandra.chavez@cinvestav.mx (ACC); nirmalkar@cinvestav.mx (KN); fernando.hernandez@cinvestav.mx (FHQ); jgmena@cinvestav.mx (JGM)

[2] Departamento de Salud, Universidad Iberoamericana, Ciudad de México, Paseo de la Reforma 880, Ciudad de México, 01219, México. Phone: +52 (55) 5950-4000 Ext. 9177; anabertha.perez@ibero.mx (APL); silvia.ramirezalto@gmail.com (SRA); cesar.hernandez@ibero.mx (CHG)

* Correspondence: cesar.hernandez@ibero.mx (CHG); Tel.: +52 (55) 5950-4000 Ext. 9177; jgmena@cinvestav.mx (JGM); Tel.: +52 (55) 5747-3800 Ext. 5328

Table S1 Relative abundance of bacterial phyla in the study subjects

Phyla	Control	Obesity	Obesity + Metabolic Syndrome	*p*-value	*q*-value
Firmicutes	56.95%	72.97%	73.34%	0.0029*	0.0345*
Bacteroidetes	36.20%	22.50%	23.43%	0.7125	1.0000
Proteobacteria	4.20%	2.80%	1.45%	0.1160	0.6960
Actinobacteria	2.32%	1.27%	1.29%	0.1667	0.8002
Tenericutes	0.11%	0.05%	0.04%	0.0893	0.6960
Cyanobacteria	0.06%	0.20%	0.07%	0.2861	0.9809
Synergistetes	0.02%	0.00%	0.00%	0.2284	0.9136
Others	0.14%	0.22%	0.37%	<0.0001	<0.0001
Total	100.00%	100.00%	100.00%	*nd*	*nd*

%, means the relative abundance. Kruskal Wallis was used to calculate the *p*-value, and the False Discovery Rate (FDR-adjusted) *q*-value. Others includes phyla such as Verrucomicrobia, Spirochaetes, and Fusobacteria. nd, not determined. *, indicates statistical significance *p*<0.05 and *q*<0.05 between Control and Obesity + Metabolic Syndrome group. See Figure 1.

Table S2 Relative abundance of bacterial orders, families and genera in the study subjects

Taxonomic category	Control	Obesity	Obesity + Metabolic Syndrome	*p*-value	*q*-value
g_*Bacteroides*	20.73%	6.47%	7.10%	<0.0001*	0.0002*
f_Ruminococcaceae	16.46%	19.16%	16.17%	0.5579	1.0000
f_Lachnospiraceae	10.96%	14.75%	17.45%	<0.0001*	0.0046*
g_*Prevotella*	6.84%	11.65%	8.85%	0.4073	1.0000
g_*Blautia*	5.09%	7.33%	8.51%	0.1430	1.0000
f_Rikenellaceae	3.77%	2.56%	2.14%	0.2519	1.0000
o_Clostridiales	2.80%	3.80%	3.86%	0.1650	1.0000
g_*Ruminococcus*	2.37%	2.09%	2.07%	0.8908	1.0000
g_*Dialister*	2.20%	1.99%	2.30%	0.0858	1.0000
g_*Streptococcus*	2.19%	0.45%	0.64%	0.0010	0.0793
g_*Coprococcus*	2.18%	4.55%	4.51%	0.0002*	0.0231*
f_Erysipelotrichaceae	1.74%	0.38%	0.36%	<0.0001*	0.0075*
o_Clostridiales	1.48%	2.40%	2.44%	0.1650	1.0000
f_Barnesiellaceae	1.45%	0.48%	0.73%	0.0357	1.0000
g_*Phascolarctobacterium*	1.45%	0.42%	0.52%	0.1064	1.0000
f_Peptostreptococcaceae	1.06%	2.07%	1.82%	0.1306	1.0000
g_*Parabacteroides*	1.03%	0.44%	0.40%	0.0008	0.0681
g_*Lachnospira*	0.99%	3.24%	3.79%	<0.0001*	0.0075*
g_*Bifidobacterium*	0.95%	0.76%	1.39%	0.9837	1.0000
g_*Eubacterium*	0.89%	0.19%	0.20%	0.0267	1.0000
g_*Collinsella*	0.89%	0.31%	0.27%	0.7422	1.0000
g_*Roseburia*	0.89%	2.72%	2.14%	0.0002*	0.0231*
f_Enterobacteriaceae	0.88%	0.21%	0.48%	0.0704	1.0000
f_Clostridiaceae	0.78%	1.38%	1.48%	0.0524	1.0000
g_*Odoribacter*	0.57%	0.30%	0.11%	0.0026	0.1729
g_*Catenibacterium*	0.56%	0.31%	0.15%	0.1058	1.0000
g_*Faecalibacterium*	0.55%	1.15%	1.19%	0.0003*	0.0290*
Others	8.26%	8.47%	8.95%	0.3679	1.0000
Total	100.00%	100.00%	100.00%	*nd*	*nd*

%, means the relative abundance. Kruskal Wallis was used to calculate the *p*-value, and the False Discovery Rate (FDR-adjusted) *q*-value. Others includes all the less abundant orders, families and genera. *nd*, not determined. *, indicates statistical significance *p*<0.05 and *q*<0.05. See Supplementary Information Figure S1. The statistical significance between pairs of groups is showed in the Supplementary Information Table S7.

Table S3 Bacterial taxa with different abundance among groups, after Benjamini-Hochberg correction

Taxonomic Hierarchy	Enriched in Group	log LDA score	*p*-value	*q*-value
k_Bacteria_p_Firmicutes_c_Clostridia_o_Clostridiales_f_Lachnospiraceae_g_Roseburia	OB	3.9411	0.0002	0.0063
k_Bacteria_p_Proteobacteria_c_Gammaproteobacteria_o_Aeromonadales_f_Succinivibrionaceae_g_Succinivibrio	OB	3.7194	<0.0001	<0.0001
k_Bacteria_p_Bacteroidetes_c_Bacteroidia_o_Bacteroidales_f_S24_7	OB	3.4940	<0.0001	<0.0001
k_Bacteria_p_Firmicutes_c_Clostridia_o_Clostridiales_f_Lachnospiraceae	OMS	4.6431	0.0001	0.0048
k_Bacteria_p_Firmicutes_c_Clostridia_o_Clostridiales_f_Lachnospiraceae_g_Lachnospira	OMS	4.1670	<0.0001	0.0011
k_Bacteria_p_Firmicutes_c_Clostridia_o_Clostridiales_f_Lachnospiraceae_g_Coprococcus	OMS	4.0980	0.0001	0.0032
k_Bacteria_p_Firmicutes_c_Clostridia_o_Clostridiales_f_Ruminococcaceae_g_Faecalibacterium	OMS	3.5611	0.0002	0.0063
k_Bacteria_p_Firmicutes_c_Clostridia_o_Clostridiales_f_Lachnospiraceae_g__Ruminococcus	OMS	3.4052	0.0001	0.0048
k_Bacteria_p_Firmicutes_c_Clostridia_o_Clostridiales_f_Veillonellaceae_g_Megamonas	OMS	3.0453	<0.0001	0.0001
k_Bacteria_p_Bacteroidetes_c_Bacteroidia_o_Bacteroidales_f_Bacteroidaceae_g_Bacteroides	CO	4.8619	<0.0001	0.0005
k_Bacteria_p_Firmicutes_c_Bacilli_o_Lactobacillales_f_Streptococcaceae_g_Streptococcus	CO	3.9944	0.0010	0.0285
k_Bacteria_p_Firmicutes_c_Erysipelotrichi_o_Erysipelotrichales_f_Erysipelotrichaceae	CO	3.9013	<0.0001	0.0017
k_Bacteria_p_Bacteroidetes_c_Bacteroidia_o_Bacteroidales_f_Porphyromonadaceae_g_Parabacteroides	CO	3.5003	0.0009	0.0272
k_Bacteria_p_Firmicutes_c_Bacilli_o_Bacillales_f_Staphylococcaceae_g_Staphylococcus	CO	3.3018	<0.0001	0.0011
k_Bacteria_p_Firmicutes_c_Bacilli_o_Turicibacterales_f_Turicibacteraceae_g_Turicibacter	CO	3.1212	<0.0001	0.0006
k_Bacteria_p_Firmicutes_c_Bacilli_o_Lactobacillales_f_Streptococcaceae_g_Lactococcus	CO	3.0615	0.0006	0.0199

The *p*-value was calculated using Linear Discriminant Analysis Effect Size (LEfSe) analysis, and the *q*-value was calculated using the Benjamini-Hochberg test. OB (Obesity), OMS (obesity + metabolic syndrome), and CO (Control) indicates the phenotypic categories. $p<0.05$ and $q<0.05$ are considered statistically significant. See Figure 4.

Table S4 Gene content prediction among groups, after FDR correction

Metabolic Pathway	Control (%)	Obesity (%)	Obesity + Metabolic Syndrome (%)	*p*-value	*q*-value
Lipid metabolism					
Lipid metabolism	0.139 ±0.011	*0.145 ±0.006	*0.145 ±0.008	0.0178	0.0292
Glycerolipid metabolism	0.400 ±0.042	0.428 ±0.027	*0.433 ±0.021	0.0017	0.0044
Synthesis and degradation of ketone bodies	0.029 ±0.008	0.032 ±0.004	*0.034 ±0.007	0.0180	0.0293
Glycerophospholipid metabolism	0.572 ±0.033	*0.600 ±0.016	0.597 ±0.020	0.0009	0.0029
Carbohydrate metabolism					
Glycolysis / Gluconeogenesis	*1.141 ±0.046	1.121 ±0.018	1.115 ±0.019	0.0138	0.0241
Pyruvate metabolism	*1.066 ±0.060	1.052 ±0.035	1.050 ±0.031	0.0098	0.0177
Amino sugar and nucleotide sugar metabolism	*1.527 ±0.105	1.441 ±0.046	1.440 ±0.062	0.0034	0.0073
Amino acid metabolism					
Alanine, aspartate and glutamate metabolism	*1.099 ±0.071	1.066 ±0.023	1.059 ±0.028	0.0010	0.0030
Metabolism of cofactors and vitamins					
Pantothenate and CoA biosynthesis	0.656 ±0.040	*0.688 ±0.022	0.686 ±0.025	0.0019	0.0047
Lipid biosynthesis proteins	*0.582 ±0.038	0.562 ±0.022	0.552 ±0.020	0.0064	0.0121
Glycan biosynthesis and metabolism					
Lipopolysaccharide biosynthesis	*0.190 ±0.113	0.110 ±0.051	0.102 ±0.048	0.0034	0.0073
Glycosaminoglycan degradation	*0.085 ±0.045	0.042 ±0.016	0.040 ±0.024	0.0000	0.0002
Metabolism of other amino acids					
Taurine and hypotaurine metabolism	*0.107 ±0.012	0.096 ±0.004	0.095 ±0.005	0.0003	0.0015
Endocrine system (Human)					
Adipocytokine signaling pathway	*0.067 ±0.022	0.047 ±0.013	0.044 ±0.012	0.0005	0.0018
Endocrine and metabolic diseases (Human)					
Type II diabetes mellitus	*0.049 ±0.004	0.047 ±0.002	0.046 ±0.002	0.0017	0.0044
Energy metabolism	*0.903 ±0.062	0.857 ±0.030	0.843 ±0.040	0.0003	0.0016

The % is reported as the mean of the relative frequencies ± standard deviation. The *p*-value was calculated using Kruskal-Wallis H-Test, and the *q*-value was calculated using the storey FDR test. Obesity, Obesity + Metabolic Syndrome, and Control indicates the phenotypic categories. An asterisk (*) indicates the highest value among groups. $p<0.05$ and $q<0.05$ are considered statistically significant. The statistical significance between pairs of groups is showed in the Supplementary Information Table S8. See Figure 5.

Table S5 Determination of the bacterial load and abundance among the studied groups

Phylum	Ct Control	Ct Obesity	Ct Obesity + Metabolic Syndrome	*p*-value
Bacterial Load[$]	16.54 ± 3.36	17.24 ± 2.22	17.09 ± 1.10	0.646
Firmicutes	21.30 ± 2.35	20.76 ± 2.83	19.52 ± 1.95	0.053*
Bacteroidetes	24.90 ± 3.75	23.81 ± 4.11	24.20 ± 3.23	0.661

Ct were determined as described in the Methods section. [$], Ct values for bacterial load using the universal primer, data are the average of three independent determinations, ± standard deviation, *p*-values were obtained using ANOVA test. *A Tukey post hoc test was made after ANOVA to identify significant differences between pair of groups; only a significant p-value of 0.046 was found when comparing Control and Obesity + Metabolic Syndrome groups.

Table S6 Post hoc test to compare among pairs of groups from all clinical characteristics

Group 1	Group 2	*p-value*
Age (years)		
CO	OMS	<0.001
CO	OB	<0.001
OMS	OB	0.779
Height (m)		
CO	OMS	0.016
CO	OB	0.011
OMS	OB	0.901
Weight (kg)		
CO	OMS	<0.001
CO	OB	<0.001
OMS	OB	0.625
BMI (kg/m^2)		
CO	OMS	<0.001
CO	OB	<0.001
OMS	OB	0.770
WC (cm)		
CO	OMS	<0.001[a]
CO	OB	<0.001[a]
OMS	OB	1.000[a]
HC (cm)		
CO	OMS	<0.001[a]
CO	OB	<0.001[a]
OMS	OB	1.000[a]
W/H ratio		
CO	OMS	<0.001[a]
CO	OB	0.003[a]
OMS	OB	1.000[a]
Fasting Glucose (mg/dl)		
CO	OMS	<0.001
CO	OB	0.110
OMS	OB	0.002
Triglycerides (mg/dl)		
CO	OMS	<0.001
CO	OB	0.882
OMS	OB	<0.001
HDL (mg/dl)		
CO	OMS	<0.001
CO	OB	<0.001
OMS	OB	0.600
LDL (mg/dl)		
CO	OMS	<0.001[a]
CO	OB	0.154[a]
OMS	OB	0.279[a]
Cholesterol (mg/dl)		
CO	OMS	0.007
CO	OB	0.972
OMS	OB	0.009
LDL-c/HDL-c ratio		
CO	OMS	<0.001[a]
CO	OB	0.006[a]
OMS	OB	0.436[a]
C/HDL-c ratio		

CO	OMS	<0.001
CO	OB	0.005
OMS	OB	0.021
SBP (mm Hg)		
CO	OMS	<0.001
CO	OB	0.006
OMS	OB	0.790
DBP (mm Hg)		
CO	OMS	0.001
CO	OB	0.092
OMS	OB	0.363

Significances were calculated using Kruskal Wallis test and Bonferroni correction (*p-value)* for non-parametric data and ANOVA with Tukey test for [a]parametric data. p<0.05 are considered statistically significant. Control – CO, Obesity – OB, Obesity + Metabolic syndrome – OMS.

Table S7 Post hoc test to compare between pairs of groups the relative abundance of bacterial orders, families and genera in the study subjects

Group 1	Group 2	*p-value*
g_Bacteroides*		
CO	OMS	<0.001
CO	OB	0.001
OMS	OB	1.000
**f_Lachnospiraceae***		
CO	OMS	<0.001
CO	OB	0.016
OMS	OB	0.972
g_Coprococcus*		
CO	OMS	0.001
CO	OB	0.001
OMS	OB	1.000
**f_Erysipelotrichaceae***		
CO	OMS	<0.001
CO	OB	0.002
OMS	OB	1.000
g_Lachnospira*		
CO	OMS	<0.001
CO	OB	0.001
OMS	OB	1.000
g_Roseburia*		
CO	OMS	<0.001
CO	OB	0.006
OMS	OB	1.000
g_Faecalibacterium*		
CO	OMS	0.001
CO	OB	0.002
OMS	OB	1.000

Significances were calculated using Kruskal Wallis test and Bonferroni correction. p<0.05 are considered statistically significant. Control – CO, Obesity – OB, Obesity + Metabolic syndrome – OMS.

Table S8 Post hoc test to compare between pairs of groups the gene content prediction from metabolic pathways of gut microbiota in all study subjects

Group 1	Group 2	*p-value*
Glycerolipid metabolism		
CO	OMS	0.001
CO	OB	0.104
OMS	OB	0.873
Synthesis and degradation of ketone bodies		
CO	OMS	0.017
CO	OB	0.217
OMS	OB	1.000
Glycerophospholipid metabolism		
CO	OMS	0.005
CO	OB	0.004
OMS	OB	1.000
Glycolysis / Gluconeogenesis		
CO	OMS	0.013
CO	OB	0.173
OMS	OB	1.000
Pyruvate metabolism		
CO	OMS	0.015
CO	OB	0.063
OMS	OB	1.000
Amino sugar and nucleotide sugar metabolism		
CO	OMS	0.005
CO	OB	0.037
OMS	OB	1.000
Alanine, aspartate and glutamate metabolism		
CO	OMS	0.001
CO	OB	0.060
OMS	OB	1.000
Pantothenate and CoA biosynthesis		
CO	OMS	0.006
CO	OB	0.009
OMS	OB	1.000
Lipid biosynthesis proteins		
CO	OMS	0.005
CO	OB	0.264
OMS	OB	0.762
Lipopolysaccharide biosynthesis		
CO	OMS	0.005
CO	OB	0.035
OMS	OB	1.000
Glycosaminoglycan degradation		
CO	OMS	<0.001
CO	OB	0.004
OMS	OB	0.737
Taurine and hypotaurine metabolism		
CO	OMS	<0.001
CO	OB	0.014
OMS	OB	1.000
Adipocytokine signaling pathway		
CO	OMS	<0.001
CO	OB	0.033
OMS	OB	1.000
Type II diabetes mellitus		
CO	OMS	0.001
CO	OB	0.104
OMS	OB	0.873
Lipid Metabolism		
CO	OMS	<0.001
CO	OB	0.097
OMS	OB	0.445
Energy Metabolism		
CO	OMS	0.033
CO	OB	0.070
OMS	OB	1.000

Significances were calculated using Kruskal Wallis test and Bonferroni correction. $p<0.05$ are considered statistically significant. Control – CO, Obesity – OB, Obesity + Metabolic syndrome – OMS.

Table S9. Alpha diversity index between pair of groups.

Group 1	Group 2	Group 1 mean	Group 1 std	Group 2 mean	Group 2 std	*p-value*
**Chao1**						
OMS	CO	769.349	101.717	583.537	87.787	0.003*
OB	CO	787.136	137.770	583.537	87.787	0.002*
OMS	OB	769.349	101.717	787.136	137.770	0.653
**Shannon**						
OMS	CO	6.562	0.379	6.324	0.459	0.090
OB	CO	6.606	0.358	6.324	0.459	0.171
OMS	OB	6.562	0.379	6.606	0.358	0.691
**Simpson**						
OMS	CO	0.973	0.010	0.967	0.017	0.519
OB	CO	0.974	0.010	0.967	0.017	0.284
OMS	OB	0.973	0.010	0.974	0.010	0.841

The results appear like mean and ± standard deviation - std. *P-value* was calculated to compares alpha diversities based on a two-sample t-test using a non-parametric methods and the default number of Monte Carlo permutations in order to find different significances among groups. Control – CO, Obesity – OB, Obesity + Metabolic syndrome – OMS. *, means significant differences between pairs of groups.

Table S10. Analysis by Beta diversity in all data set and in a paired age sub-sample.

	Sample size	Groups	Test statistic	*p-value*
Unweighted				
All CO, OB and OMS participants	67	3	0.20	0.01
Sub-sample controlled by age	9	3	0.32	0.02
Weighted				
All CO, OB and OMS participants	67	3	0.08	0.01

P-value was calculated to compares beta diversities using a distance matrix as the primary input and mapping file. ANOSIM method such a non-parametric test and the default number of permutations in order to find different significances among groups. Control – CO, Obesity – OB, Obesity + Metabolic syndrome – OMS. $p < 0.05$ is considered statistically significant.

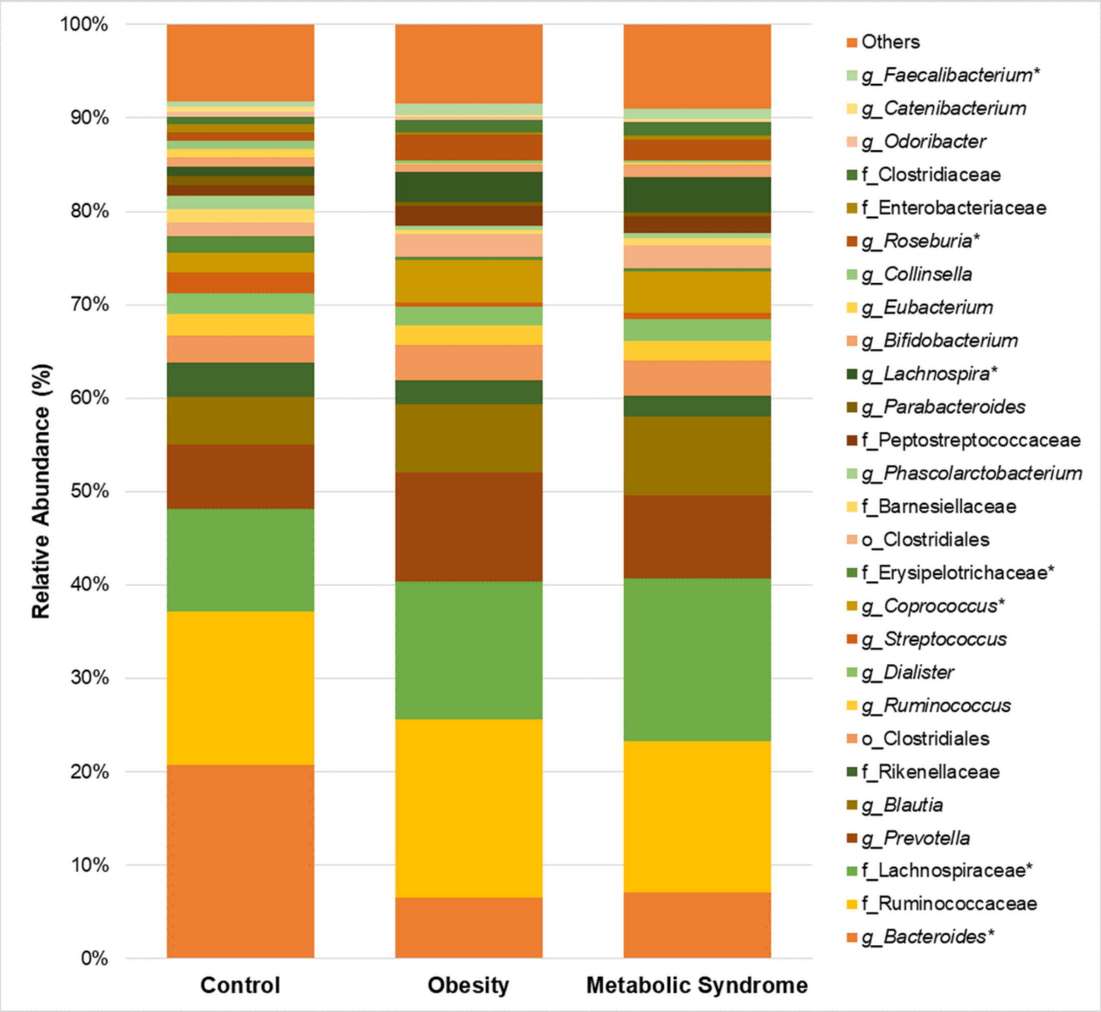

Figure S1. Bacterial diversity in Control, Obesity, and Obesity + Metabolic syndrome. The figure shows bar charts of relative abundance for relevant bacterial orders, families and genera, present in the three phenotypic categories. Each taxonomic hierarchy is identified by colors as indicated beside the bars. An asterisk shows bacteria with significant change in the relative abundance (see results).

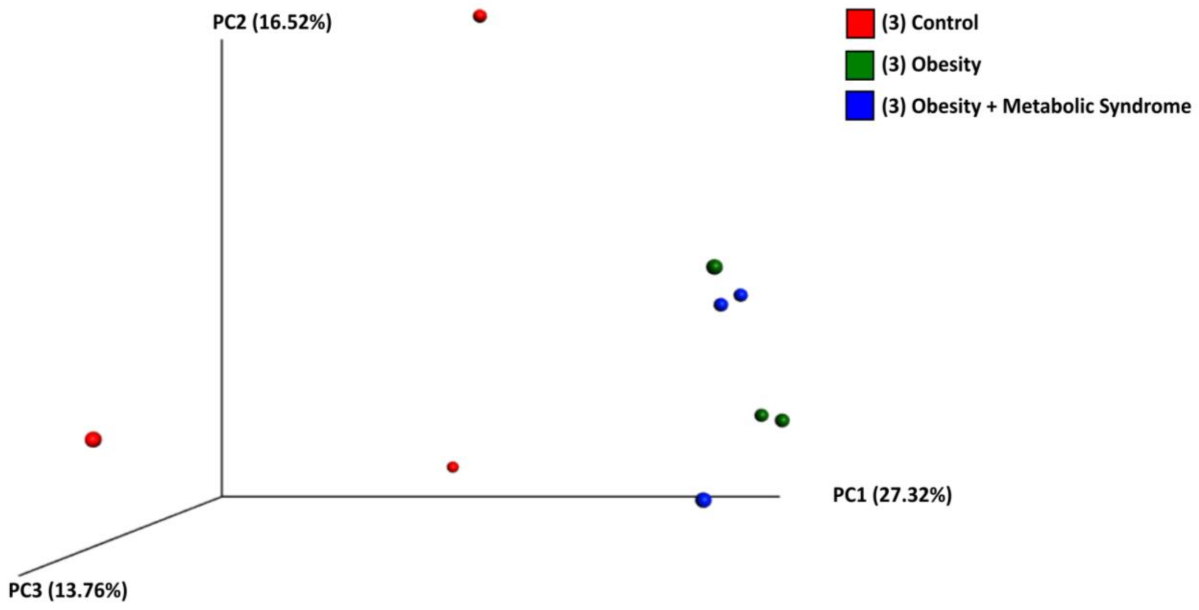

Figure S2. Bacterial beta diversity. The Figure shows a three-dimensional scatter plot, generated using principal coordinates analysis (PCoA) from Unweighted UniFrac analyses, showing the distance of microbial communities among women with normal weight (red color spheres), women with obesity (green color spheres), and obesity + metabolic syndrome (blue color spheres). Each group is identified by colors as indicated on the right side of the figure. P-value was calculated using ANOSIM method to compare beta diversities using a distance matrix as the primary input and mapping file. Control, Obesity, and Obesity + Metabolic syndrome have significant differences (*p-value* 0.02). See Table S10.

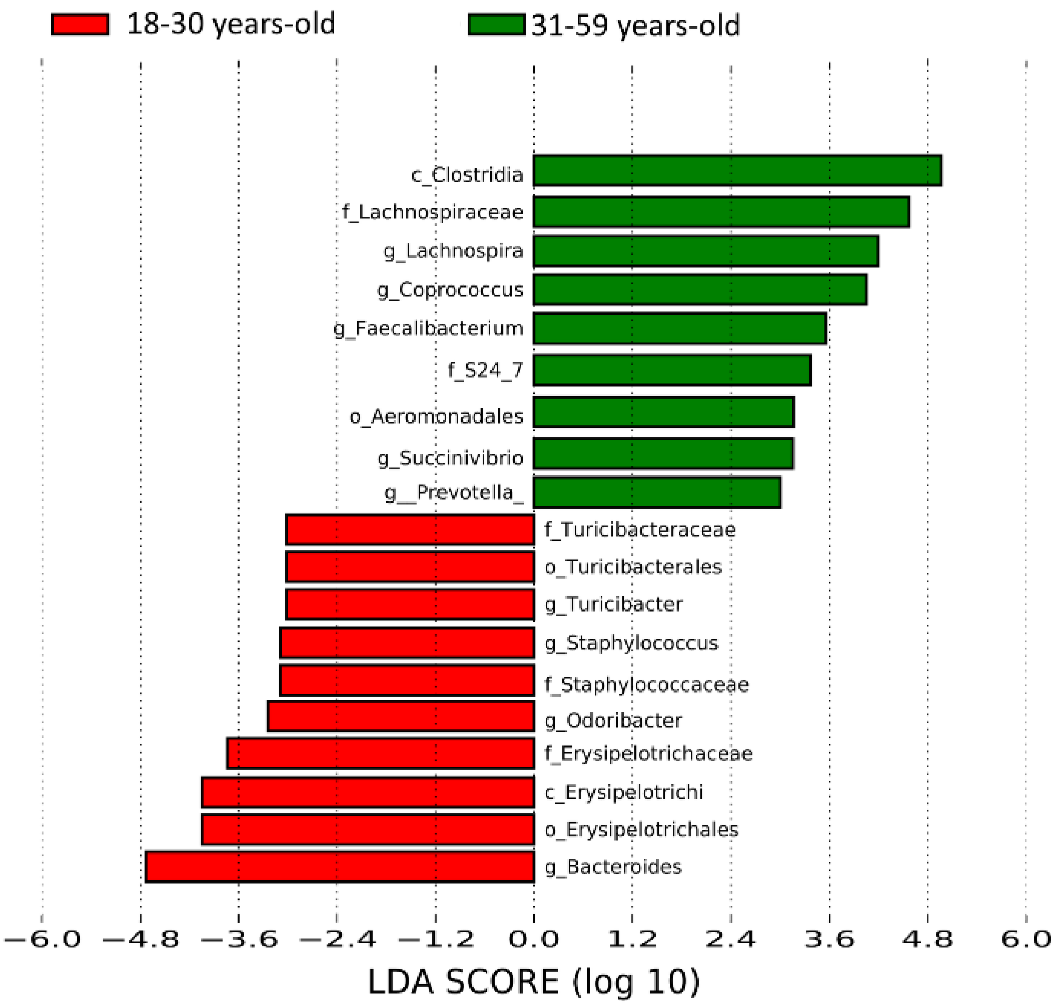

Figure S3. Linear discriminant analysis Effect Size (LEfSe) for the bacterial communities. The LEfSe plot shows enriched bacterial families and genera significantly associated with two age group categories. Ten bacteria were enriched in the 18-30 years-old group (red) and nine bacteria in the 31-59 years-old group (green). The LDA score or Effect size is shown at logarithmic scale underneath the bars. Each group is identified by color on top of the figure.

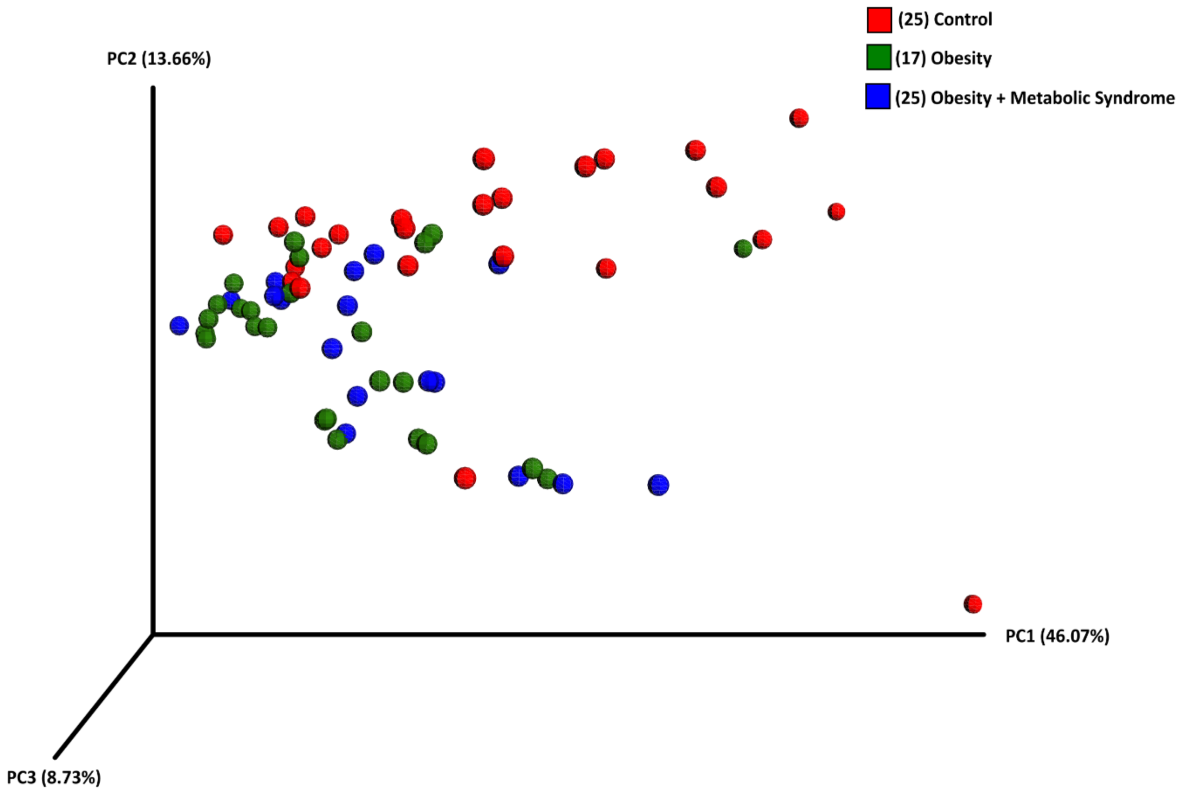

Figure S4. Bacterial beta diversity. The Figure shows a three-dimensional scatter plot, generated using principal coordinates analysis (PCoA) from Weighted UniFrac analyses, showing the distance of microbial communities among women with normal weight (red color spheres), women with obesity (green color spheres), and obesity + metabolic syndrome (blue color spheres). Each group is identified by colors as indicated on the right side of the figure. *p*-value was calculated using ANOSIM method to compare beta diversities using a distance matrix as the primary input and mapping file. There are not significant differences among groups (*p*-value 0.01).

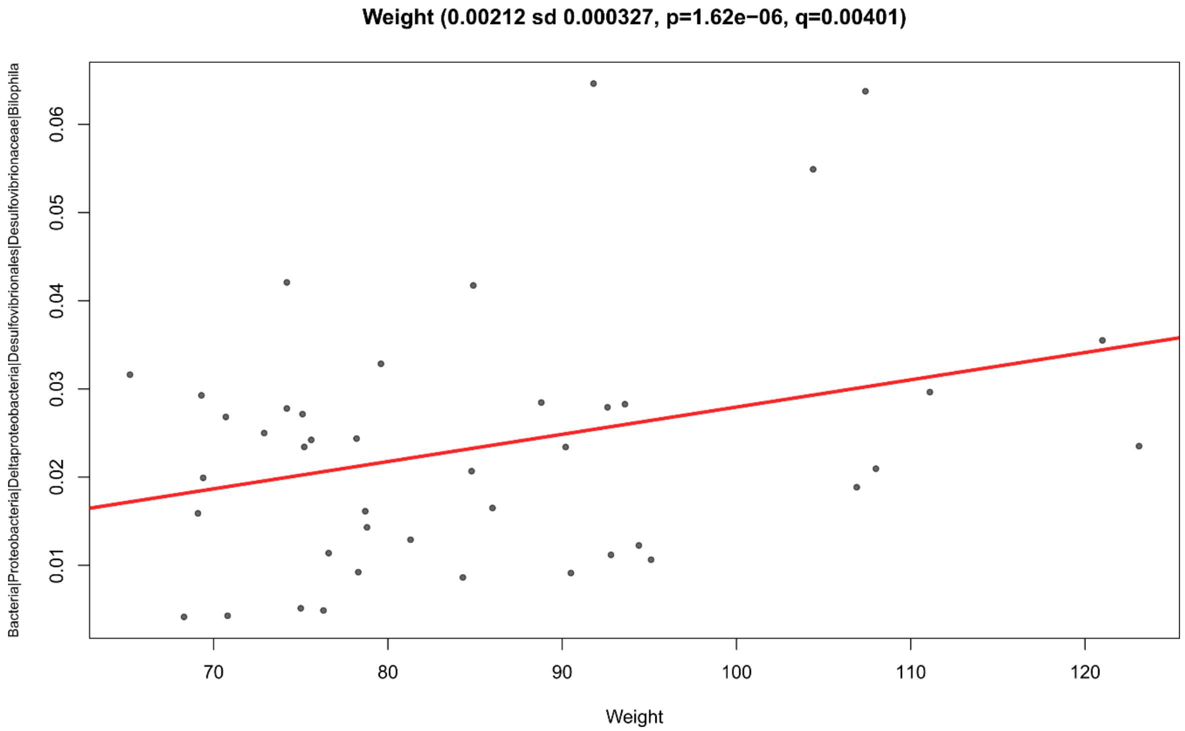

Figure S5. Multivariate linear associations of clinical metadata and bacterial relative abundance in all participants. Scatter plot explains the significant association of body weight with *Bilophila* as described in Materials and Methods. y-axes show the relative abundance of gut microbiota; x-axes show the clinical metadata. Numerical data on top of graphic are Coefficient (positive coefficient shows positive association between metadata and gut microbiota), *sd*—standard deviation; *e*—times 10 is raised to the power of; *p*-values, and FDR corrected *q*-values which are assigned by MaAsLin (v0.0.4).

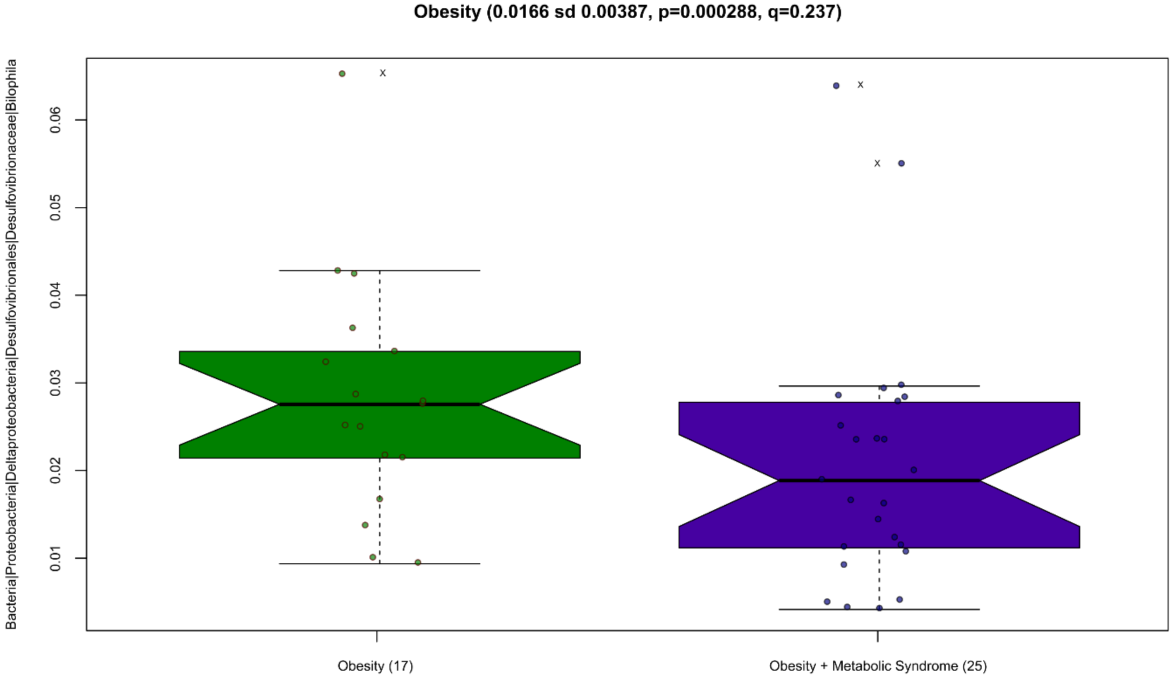

Figure S6. Multivariate linear associations of clinical metadata and bacterial relative abundance with OB and OMS. Scatter plot explains the significant association of phenotypic category with *Bilophila* as described in Materials and Methods. y-axes show the relative abundance of gut microbiota; x-axes show the clinical metadata. Numerical data on top of graphic are Coefficient (positive coefficient shows positive association between metadata and gut microbiota), *sd*—standard deviation; *e*—times 10 is raised to the power of; *p*-values, and FDR corrected *q*-values which are assigned by MaAsLin (v0.0.4).
